# Skin bacterial diversity is higher on lizards than sympatric frogs in tropical Australia

**DOI:** 10.7717/peerj.5960

**Published:** 2018-11-14

**Authors:** Chava L. Weitzman, Karen Gibb, Keith Christian

**Affiliations:** 1 Independent Researcher, Millburn, NJ, United States of America; 2 Department of Biology, University of Nevada—Reno, Reno, NV, United States of America; 3 Research Institute for the Environment and Livelihoods, Charles Darwin University, Casuarina, Northern Territory, Australia

**Keywords:** Cutaneous microbiome, Illumina MiSeq, Australia, Reptile, Amphibian, Frog skin bacteria, Lizard skin bacteria

## Abstract

Animal skin acts as a barrier between the organism and its environment and provides the first line of defense against invading pathogens. Thus, skin surfaces harbor communities of microbes that are interacting with both the host and its environment. Amphibian skin bacteria form distinct communities closely tied to their host species, but few studies have compared bacterial communities between amphibians and other, non-amphibian sympatric animals. Notably, skin microbes on reptiles have gained little attention. We used next-generation sequencing technology to describe bacterial communities on the skin of three lizard species and compared them to bacteria on six cohabiting frog species in the Northern Territory of Australia. We found bacterial communities had higher richness and diversity on lizards than frogs, with different community composition between reptiles and amphibians and among species. Core bacteria on the three lizard species overlapped by over 100 operational taxonomic units. The bacterial communities were similar within species of frogs and lizards, but the communities tended to be more similar between lizard species than between frog species and when comparing lizards with frogs. The diverse bacteria found on lizards invites further questions on how and how well reptiles interact with microorganisms through their scaly skin.

## Introduction

Microbes, though ubiquitous, form distinct communities in and on different surfaces. The rise in animal–microbiome studies has focused on gut microbes and their roles and associations with host trophic level and evolutionary history, with a large focus on mammalian hosts (Colston & Jackson, 2016). The importance of microbes in other contexts, however, has excited an influx of ecological studies moving away from mammals and digestion. On frogs, for instance, sympatric species can have divergent microbiome communities, which can also differ from the communities found in their surrounding environments (McKenzie et al., 2012; Kueneman et al., 2014; Walke et al., 2014; Bates et al., 2018), although there is nevertheless an environmental component (Hughey et al., 2017). Further research has even found changing bacterial communities across amphibian ontogeny (Kueneman et al., 2014; Warne, Kirschman & Zeglin, 2017; Bates et al., 2018).

On the skin of animals, microbes can play a functional role in host immunity. Pathogenic fungi are detected in multiple animal species, causing diseases such as white nose syndrome in bats (Blehert et al., 2009), snake fungal disease (Lorch et al., 2016), and chytridiomycosis of salamanders (Martel et al., 2013) and frogs (Fisher, Garner & Walker, 2009). On amphibian skin, some members of the bacterial community can inhibit pathogenic fungi (Harris et al., 2006; Lauer et al., 2007). Just as infected and healthy individuals may have differing microbial communities, those communities also change with the onset of infection (Jani & Briggs, 2014). Studies have begun to address the anti-fungal capacities of microbes in other systems of fungal diseases. For instance, Hill et al. (2018) used culturing techniques to find bacterial isolates that inhibit the fungus causing disease in North American snakes.

As a mucosal surface, properties of amphibian skin have strong interactions with the microbes present and play a part in maintaining homeostasis and immunity (Rollins-Smith & Woodhams, 2012; McFall-Ngai et al., 2013). The skin mucosal surface is also assumed to support a diverse microbial community due to its moist environment and ties to host health (Rollins-Smith & Woodhams, 2012). For similar reasons, bacterial communities have been studied on reptilian mucosal surfaces, such as the nasal cavity, cloaca, and gut (Troyer, 1982; Mackie et al., 2004; Santoro et al., 2006; Ordorica et al., 2008; Yuan et al., 2015). Microbes on the skin of reptiles, however, are understudied (Colston & Jackson, 2016), including how reptilian skin microbiomes diverge from those on sympatric organisms. We presume that without the energy source provided to bacteria from mucus, there are inherent differences in how reptiles interact with bacteria on their skin versus along mucus membranes, and how skin bacterial communities interact with their reptilian hosts compared with those on amphibian hosts.

In a separate study (Christian et al., 2018), we analyzed cutaneous microbial patterns among species, sites, and ecological habit (terrestrial, arboreal) on frog species in the Northern Territory of Australia. Here, we expand our analyses to include nearby lizards. We sampled bacteria from the skin of three lizard species and six frog species at a single site to compare bacterial communities across distantly related, sympatric ectothermic tetrapods. After describing the communities on lizards, we test two hypotheses: (1) Bacteria on lizard skin form distinct assemblages from sympatric frog species; (2) frogs have a closer relationship to their skin microbes, thus, frog skin bacterial communities will be less variable within species than those on lizard species.

## Methods

### Sample collection

Approval to sample frogs and lizards was granted by the Charles Darwin University Animal Ethics Committee (project A14012). We collected skin swab samples from three lizard (*Carlia gracilis, Gowidon temporalis, Hemidactylus frenatus*) and six frog (*Limnodynastes convexiusculus, Litoria caerulea, Lit. nasuta, Lit. rothii, Lit. rubella, Rhinella marina*) species on or near the campus of Charles Darwin University in Casuarina, Northern Territory, Australia in 2015. Ten lizards and 20 frogs were swabbed per species. Individuals were caught by hand, rinsed twice with 100 mL 0.45 µm high purity water (Culp, Falkinham III & Belden, 2007; Lauer et al., 2007) and swabbed with a sterile synthetic swab (microRheologics FLOQSwab). Swabbing consisted of 30 strokes to represent the entire body excluding the head and cloaca, including 10 strokes around the main body region (four dorsal, four ventral, and one each side) and five strokes on each limb (front and back of foot, front and back of leg, and axial region). Swabs were placed on ice in the field before being stored at −20 °C until DNA extraction. New gloves were worn for handling of each swabbed animal.

### DNA extraction and sequencing

DNA from each swab was extracted using the Qiagen DNeasy Blood and Tissue Kit (QIAGEN, Valencia, CA) following the tissue extraction protocol. DNA was quantified with a Nanodrop spectrophotometer, and 200 ng dried DNA was sent to Molecular Research DNA (http://www.mrdnalab.com, Shallowater, TX, USA) for sequencing.

DNA samples were sequenced using an Illumina MiSeq platform targeting the V4 variable region of the 16S rRNA gene with F515/R806 primers (Caporaso et al., 2011) and barcodes attached to the forward primer. PCR was run with the HotStarTaq Plus Master Mix kit (QIAGEN) with the following conditions: initial denaturation at 94 °C for 3 min; 28 cycles of 94 °C for 30 s, 53 °C for 40 s, 72 °C for 1 min; and a final elongation at 72 °C for 5 min. PCR product was checked for amplification success on 2% agarose gel. Samples were pooled in equal proportions and purified with Ampure XP beads. DNA libraries were prepared following the Illumina TruSeq protocol. Sequence filtering and downstream analyses were conducted on raw MiSeq data from BaseSpace.

### Sequence filtering and OTU calling

Using QIIME v. 1.9.1 (Caporaso et al., 2010), barcodes were extracted, sequences were demultiplexed, and we filtered out sequences <289 bp in length, as well as sequences with ambiguous base calls or more than one expected base error. Quality filtering passed approximately 93% of sequences. After removal of chimeras, operational taxonomic units (OTUs) were clustered based on 97% similarity, and taxonomy was assigned using uclust (Edgar, 2010), with default parameters for open reference OTU picking. Only OTUs identified as Bacteria were kept, additionally excluding chloroplast and mitochondrial sequences. As the focus of this study was to describe lizard skin bacterial communities and compare them with those on frogs, we randomly chose ten samples from each frog species to include in this study. OTUs represented in only one sample and those that accounted for less than 0.01% of the total sequence abundance were excluded in further analyses. Data were rarefied to 13,000 reads per sample.

### Description of lizard skin bacterial communities and comparison with local amphibians

To detect associations between bacteria and their hosts, we identified core OTUs, i.e., those found in most samples per species, with thresholds of 90% and 100% presence.

Diversity metrics, distance matrices, and non-metric multidimensional scaling (NMDS), were all calculated in QIIME, while statistical analyses were conducted in the programming language R (v. 3.4.4; R Development Core Team, 2015). OTU richness, Shannon’s diversity metric, and Faith’s phylogenetic diversity were separately compared among species with Kruskal–Wallis tests. Post-hoc Dunn’s tests were performed using the FSA and dunn.test packages (Dinno, 2015; Ogle, 2018) with *P*-values adjusted with a Benjamini Hochberg correction. To assess the variability of the lizard bacteria compared to those on frogs, we visualized the communities using NMDS with a UniFrac distance matrix (Lozupone & Knight, 2005) weighted for OTU abundance. We further used this distance matrix to compare species’ bacterial communities with permutational analysis of variance (PERMANOVA). PERMANOVAs were performed using the adonis function in the vegan package (Oksanen et al., 2018) with 999 permutations among all species and between frogs and lizards. Pairwise PERMANOVAs between species were conducted with 999 permutations using the pairwise.perm.manova function in the RVAideMemoire package (Hervé, 2018) with a Benjamini Hochberg adjustment of *P*-values.

## Results

**Figure 1:**
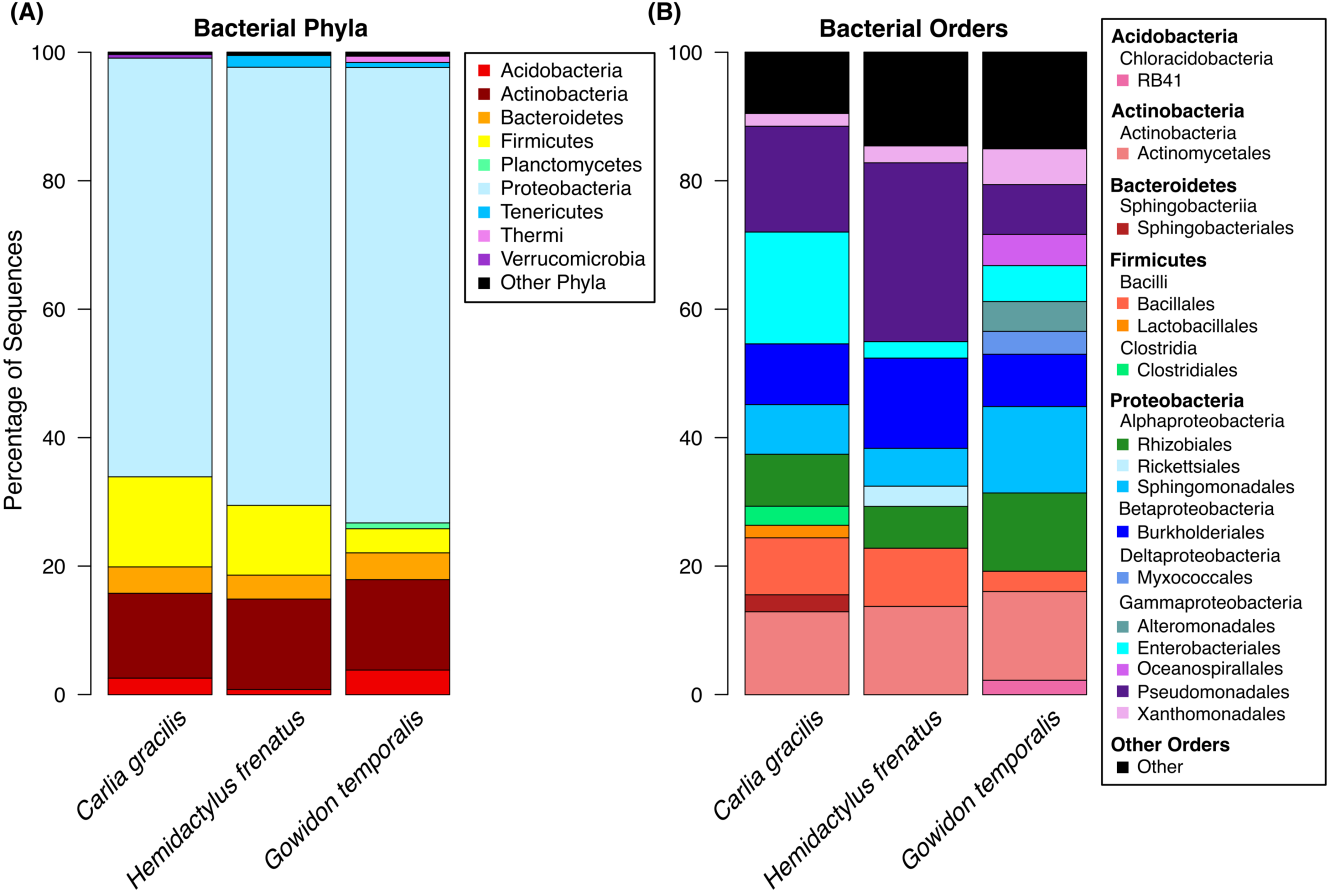
Bacterial taxa sequenced from the skin of three lizard host species in the Northern Territory of Australia. (A) Bacterial phyla representing at least 0.5% of the sequence abundance for each host species. (B) Bacterial orders representing at least 2% of the relative abundance for each species. Data subset to 13,000 sequences per sample.

A total of 738 OTUs were detected on the three lizard species sampled after data filtering (without rarefaction). Bacterial communities were dominated by few taxa, largely represented by the bacterial phyla Proteobacteria, Actinobacteria, Firmicutes, and Bacteroidetes. The most abundant orders on lizard skin included Pseudomonadales, Actinomycetales, Burkholderiales, Sphingomonadales, Rhizobiales, and Enterobacteriales along with 10 others amounting to at least 2% of the sequences on a host species (Fig. 1).

**Figure 2:**
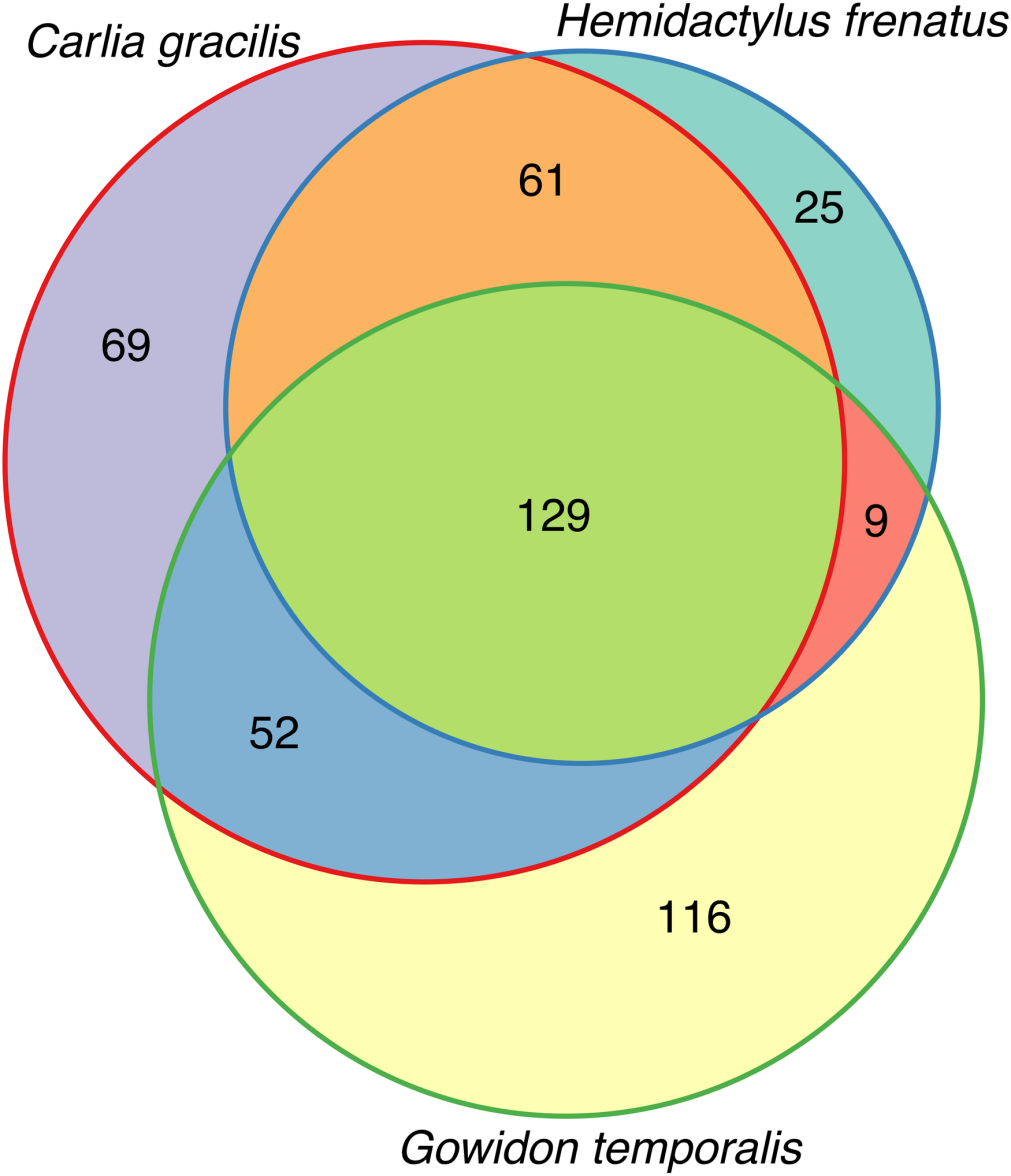
Venn diagram of core OTUs (100% presence) on three lizard species.

Lizards had hundreds of core OTUs (100% prevalence), accounting for 32–44% of the total OTUs detected per species (Table 1), and all three host species shared 129 core OTUs (Fig. 2). Percent OTU richness from core OTUs was approximately double on lizards versus frogs, excluding *Lim. convexiusculus* (51% richness from core OTUs). A similar pattern occurred with a core cut-off of 90%, with lizard core OTUs accounting for 47–62% of OTU richness, *Lim. convexiusculus* core OTUs representing 67% of its richness, and the remaining frog species with core communities accounting for 22–32% richness.

**Table 1:** Total OTU richness and core OTUs per host species. Total OTU count in 13,000 sequences per sample (unrarefied values in parentheses). Core OTUs at 100% prevalence cut-off.

**Species**	**Total OTUs**	**Core OTUs**	**% Core**
Lizards			
*Carlia gracilis*	707 (726)	311	44.0%
*Hemidactylus frenatus*	710 (716)	224	31.5%
*Gowidon temporalis*	691 (721)	306	44.3%
Frogs			
*Limnodynastes convexiusculus*	716 (725)	362	50.6%
*Litoria caerulea*	680 (725)	109	16.0%
*Lit. nasuta*	731 (737)	146	20.0%
*Lit. rothii*	696 (714)	121	17.4%
*Lit. rubella*	633 (682)	121	19.1%
*Rhinella marina*	676 (716)	144	21.3%

**Figure 3:**
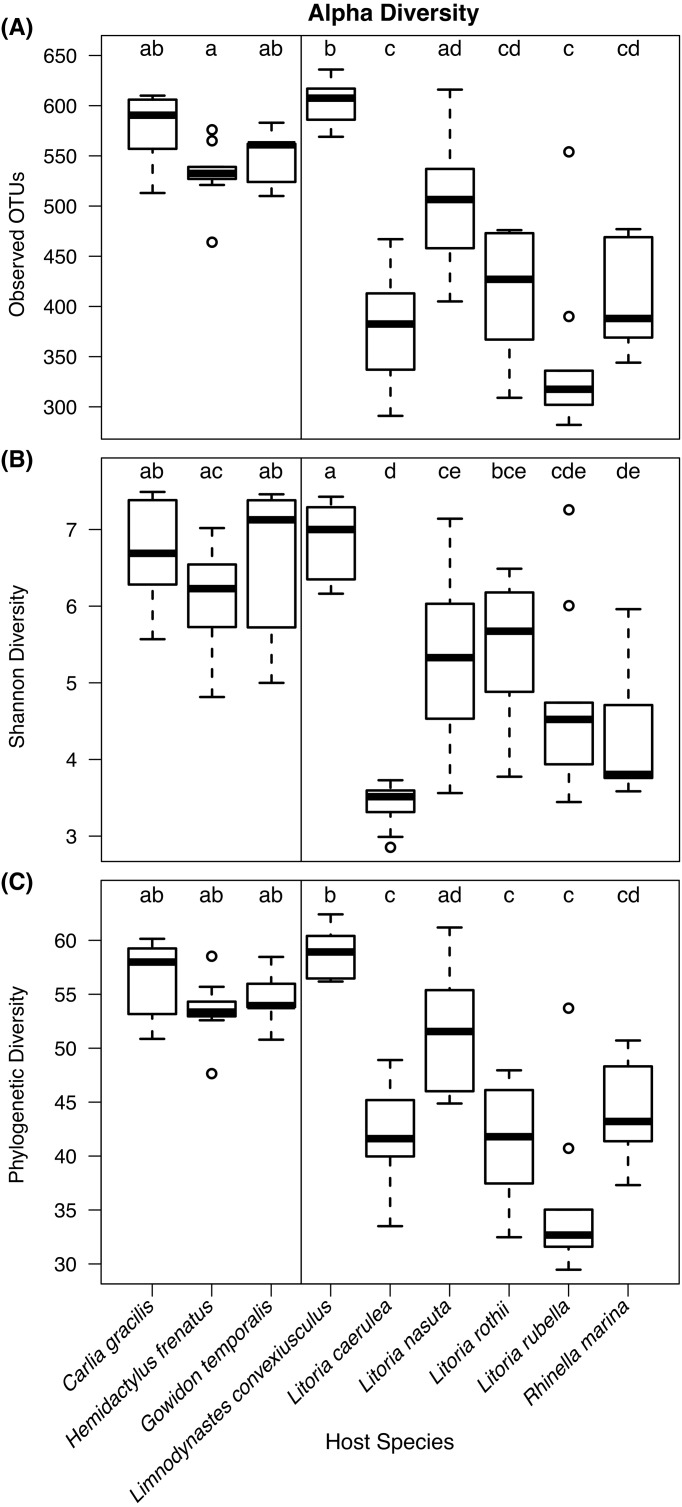
Boxplots of alpha diversity metrics by host species. (A) Observed OTU richness, (B) Shannon diversity, and (C) Faith’s phylogenetic diversity. Boxplots indicate the median, interquartile range, reasonable range of the data, and outliers. Vertical line separates lizard species on the left from frog species on the right. Letters above plots indicate significant differences by post-hoc Dunn’s tests with Benjamini Hochberg adjusted *P* < 0.05.

Kruskal–Wallis tests detected significant differences in observed OTU richness (*χ*^2^ = 69.80, *df* = 8, *P*  < 0.00001), Shannon diversity (*χ*^2^ = 58.72, *df* = 8, *P* < 0.00001), and phylogenetic diversity measures (*χ*^2^ = 68.37, *df* = 8, *P* < 0.00001) among species. From pairwise Dunn’s tests, alpha diversity metrics were found to be higher in lizard species than most frog species (Fig. 3; pairwise *P*-values in Supplementary Information). In most comparisons, the frog species *Lim. convexiusculus* had higher richness and diversity than the other amphibians, similar to those found on lizards (Fig. 3).

**Figure 4:**
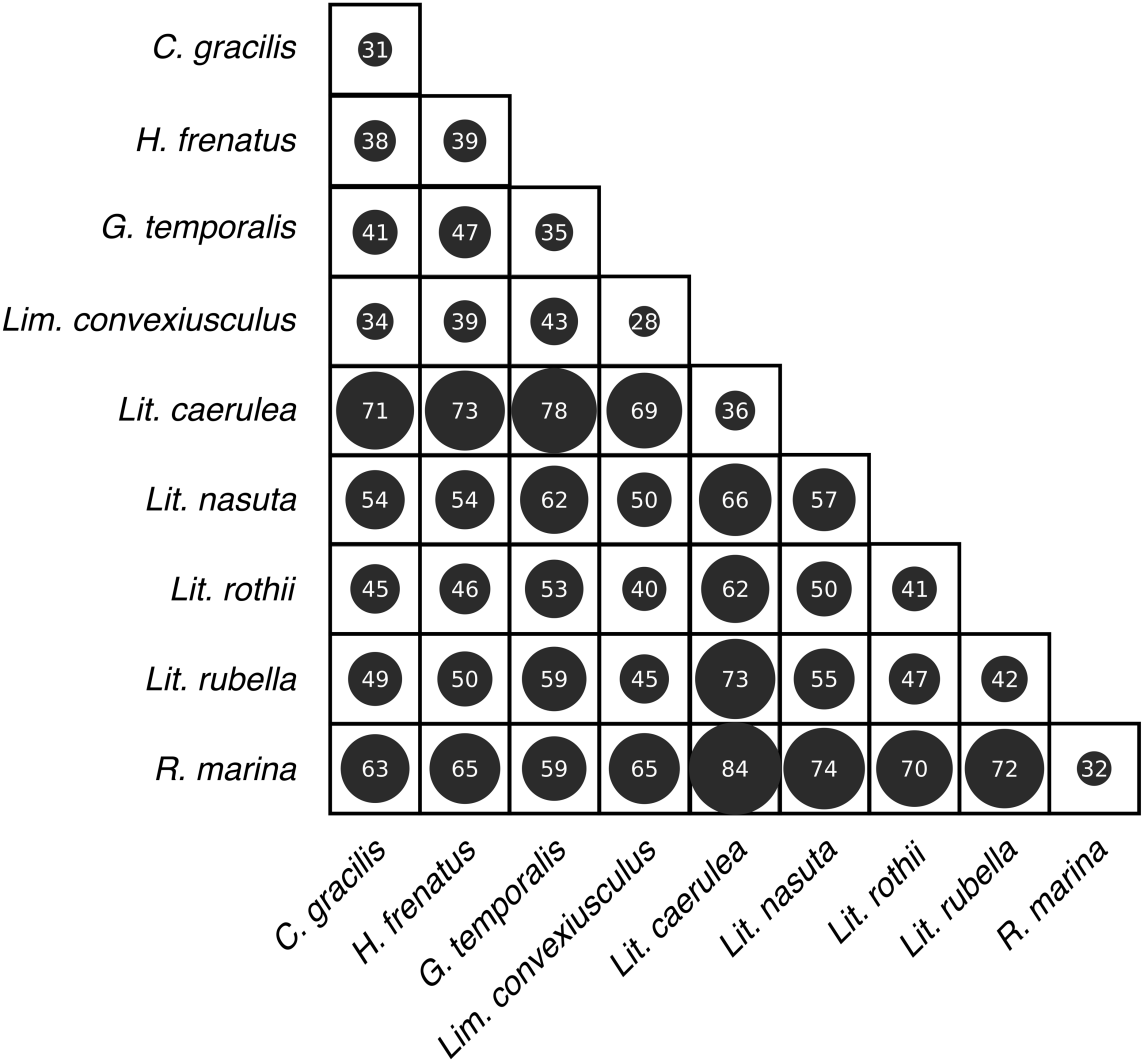
Average weighted UniFrac distances (100×) within and between species. Larger circles and numbers represent greater dissimilarities. Data subset to 13,000 sequences per sample.

**Figure 5:**
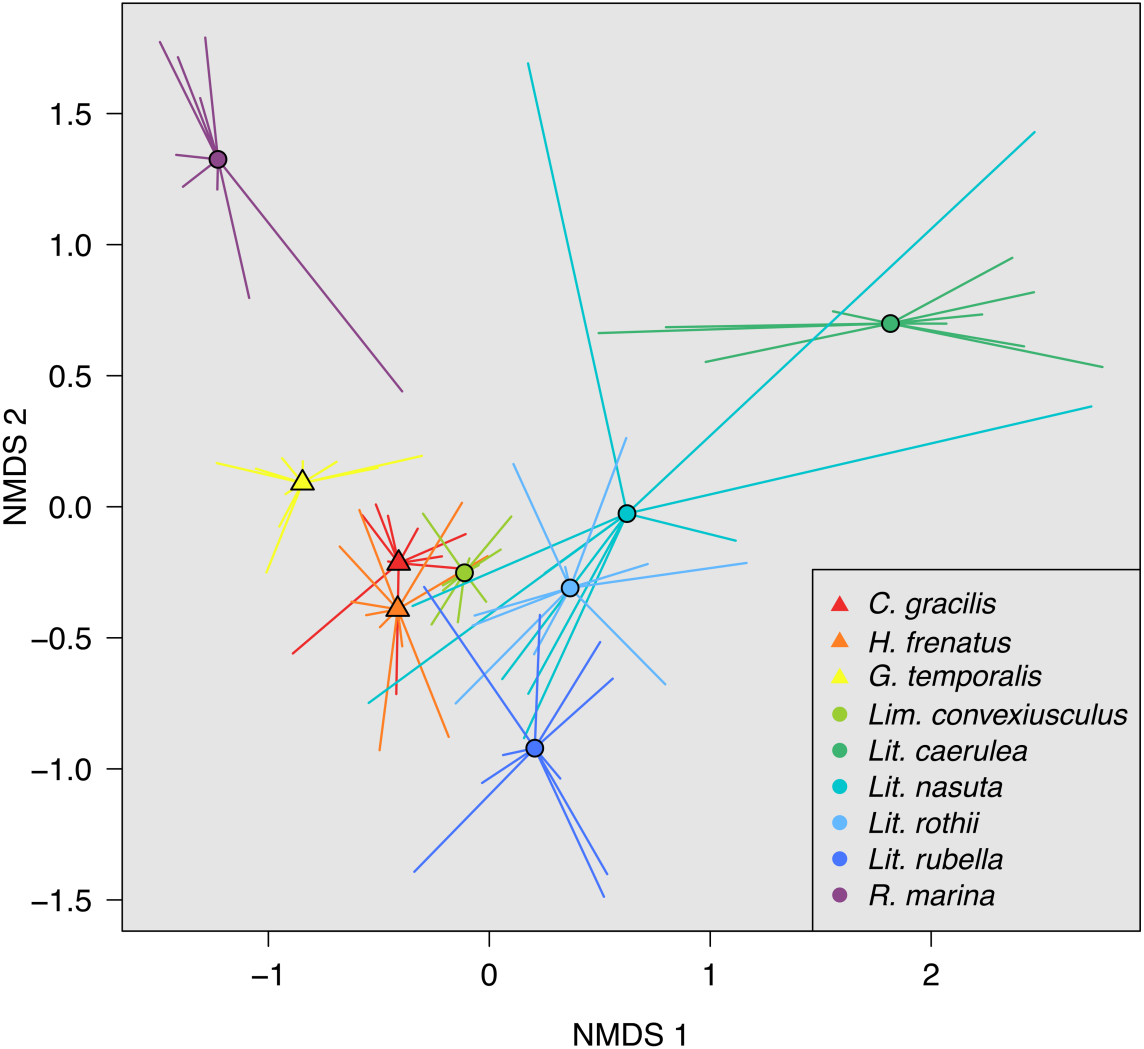
Non-metric multidimensional scaling (NMDS) of weighted UniFrac distances. Mean NMDS coordinates for each species are denoted by symbols, with vectors connecting means to individual points. Data color-coded by species. Lizards = triangles, Frogs = circles. Stress = 0.143.

Skin bacterial communities on the lizard species had similar average UniFrac distances within-species to those found on amphibians, though *Lit. nasuta* frogs had intraspecific UniFrac distances much higher than all other species (Fig. 4). Each of the three lizard species had communities that differed most from those on *Lit. caerulea*, *R. marina*, and *Lit. nasuta*. Distances between lizard species were generally lower than between-frog-species distances, i.e., lizard species’ bacterial communities were more similar than those on most pairs of frog species (Figs. 4, 5). PERMANOVAs found significant community differences among species (*R*^2^ = 0.55, *P* = 0.001) and between reptiles and amphibians (*R*^2^ = 0.09, *P* = 0.001). Pairwise PERMANOVAs detected significantly different bacterial communities between all species (*P* < 0.015 each after Benjamini Hochberg correction), except between the two frog species *Lit. nasuta* and *Lit. rothii* (*P* = 0.265).

## Discussion

We sampled skin bacteria on three lizard species on or near the campus of Charles Darwin University and found diverse communities on these reptiles. Similar to communities on sympatric frog species (Christian et al., 2018; supplemental material), the skin bacteria of lizards were largely represented by the orders Pseudomonadales, Actinomycetales, Burkholderiales, Sphingomonadales, Rhizobiales, and Enterobacteriales. These orders comprised approximately 61–72% of the total OTU abundance, with many additional taxa present. These data represent bacteria found on the entire body of the animals swabbed, excluding the head, tail (for lizards), and cloaca.

The bacterial richness on the species we sampled was approximately 700 OTUs. Lizards generally had richer core bacterial communities than frogs, with much overlap in lizard core OTUs. Eighty-nine percent of the core microbiome of non-native common house geckos (*H. frenatus*) was also core to one or both of the other lizards, making this core less unique than the other two lizard species. The wide distribution of this gecko due to anthropogenic translocation offers an opportunity to study geographic variation in its cutaneous microbiota. The core microbiome may include taxa that have co-evolved with the host to aid in the regulation of physiological processes and pathogen interactions (Harris et al., 2006; Turnbaugh & Gordon, 2009; Grice & Segre, 2011; Apprill et al., 2014; Loudon et al., 2014). Nearly 60% overlap in core bacteria on the native lizard species invites further research determining the functional roles of skin microbes on reptiles and the strength of the environment in shaping skin bacterial communities.

Due to the strong relationship frogs have with their cutaneous microbiota, we predicted that the bacterial communities between frogs and lizards, and between species, would be different. We also predicted less variability on and among frogs than lizards. While we did find different bacterial community assemblages among lizard species, and between all lizard species and sympatric frogs, we found surprisingly high bacterial diversity on lizards. Lizards had greater microbial richness and diversity than most of the six frog species sampled. The strong exception was the marbled frog, *Lim. convexiusculus*, which in many ways had communities more similar to those on lizards than the other frog species (Figs. 3–5). Accordingly, UniFrac distances between lizards and *Lim. convexiusculus* were, on average, lower than the distances between bacteria on lizards or *Lim. convexiusculus* and all other frog species. The greater diversity and richness of bacteria on lizards could signify a looser association of lizards with their skin microbes, allowing for more transient taxa. However, a higher percentage of OTUs were core on lizards, and intra- and inter-specific communities on lizards were unexpectedly similar; neither of these results support the “transient taxa” explanation. An alternate explanation is that a loose interaction between lizard hosts and their skin microbes might allow more bacterial types to survive, and similarities among lizards’ skin allow similar bacteria to thrive.

Importantly, the lizards we sampled use different microhabitats, with some overlap with the frog species sampled: *C. gracilis* (slender rainbow skinks) live on the ground and in leaf litter, as do *Lim. convexiusculus* (marbled frogs), *Lit. nasuta* (striped rocket frogs), and *R. marina* (cane toads); *H. frenatus* (common house geckos) stay mostly on buildings, where *Lit. caerulea* (green tree frogs) and *Lit. rubella* (desert tree frogs) are also found; and *G. temporalis* (swamplands lashtail dragons) reside mostly in trees, along with *Lit. rothii* (Roth’s tree frogs). Thus, similarities among lizard skin bacteria cannot be explained by environmental similarities, but rather, features of lizard skin may result in similar microbiomes regardless of the environment. This poses the questions: If additional lizard species were sampled, would they also possess similar bacterial communities? Is there an environmental, geographic, or phylogenetic threshold beyond which skin bacteria are no longer similar? And what anatomical or physiological characteristics affect or support the skin bacterial community composition that we find on lizards?

In addition to interacting with other members of the microbial community, skin bacteria may be affected by antimicrobial peptides (AMPs) secreted by the host (Conlon, 2011a; Holden et al., 2015). Though reptiles produce diverse AMPs, there is minimal information on the presence of AMPs on lizard epidermis outside of the context of wounding (Van Hoek, 2014; Conlon, 2015). One study found a single AMP expressed in the skin of healthy *Anolis* lizards, with greater AMP diversity elsewhere in the body (Dalla Valle et al., 2013). The scales on lizard skin provide a barrier to infection, therefore, lower AMP expression and higher bacterial diversity might not affect the health of the animal and could allow for high bacterial diversity. While AMPs are poorly studied and may be uncommon on lizard skin, those produced by frogs are more prevalent and better understood, but the importance of AMPs as an innate immune response in frogs is still unclear (Conlon, 2011b).

In contrast with the influx of research on bacterial interactions with amphibian chytridiomycosis, much less data are presently available on interactions between reptilian skin bacteria and pathogens. As in other taxa, reptiles are susceptible to skin diseases. Recently, Allender et al. (2018) found distinct microbial communities on eastern massasauga rattlesnakes (*Sistrurus catenatus*) in the United States with and without snake fungal disease. A study on skin bacteria from timber rattlesnakes (*Crotalus horridus*) and black racers (*Coluber constrictor*) from the U.S. found isolates capable of inhibiting the fungal pathogen *Ophidiomyces ophiodiicola* (Hill et al., 2018). Additional fungi, identified as the *Chrysosporium* anamorph of *Nannizziopsis vriesii* (CANV) and *Chrysosporium* spp., also cause sometimes fatal skin infections in multiple reptilian taxa, including lizards, crocodilians, and tuatara. While these infections are predominantly found in captive animals, they are also present on wild reptiles (Sigler, Hambleton & Paré, 2013). Future work should focus on interactions between microbiome community members (pathogenic and non-pathogenic) and the roles bacteria play in immunity in these reptilian systems.

## Conclusions

In the Northern Territory of Australia, sympatric frog and lizard species had distinct bacterial communities on their skin. Lizard bacteria were more diverse and less variable between species than the bacterial microbiomes on frog skin, suggesting that these are not transient taxa, but the relative influences of the environment and interactions with the host are not known. A study on lizard skin microbiota using more host species would allow us to determine whether the pattern of highly rich, diverse, and yet in many ways similar bacterial communities we found on lizards continues in a wider geographic and host-species range.

##  Supplemental Information

10.7717/peerj.5960/supp-1 Data S1 Converted biom table with consensus lineages from open reference OTU picking File contains bacterial OTU data on ten individuals per species swabbed. Data filtered to include OTUs representing at least 0.01% sequence abundance and subset to 13,000 sequences per sample.

10.7717/peerj.5960/supp-2 Data S2 Metadata for OTU table, including host species, barcodes attached for sequencing, and swab date

10.7717/peerj.5960/supp-3 Table S1 Results from pairwise post-hoc Dunn’s tests and PERMANOVAs Contains one sheet per set of pairwise analysis results: OTU richness, Shannon diversity, Phylogenetic diversity, and PERMANOVAs. Adjusted p-value is provided for each pairwise comparison.

## Additional Information and Declarations

### Data Availability

The following information was supplied regarding data availability: Sequences from this study are openly available on figshare, DOI: 10.6084/m9.figshare.7072511. OTU data used in analyses and its associated metadata are provided in Data S1–S2. Pairwise post-hoc *p*-values are provided in Table S1.
